# Translation of TRO40303 from myocardial infarction models to demonstration of safety and tolerance in a randomized Phase I trial

**DOI:** 10.1186/1479-5876-12-38

**Published:** 2014-02-07

**Authors:** Sophie Le Lamer, Stéphanie Paradis, Hidayat Rahmouni, Corinne Chaimbault, Magali Michaud, Marcel Culcasi, Jean Afxantidis, Mathilde Latreille, Patrick Berna, Alain Berdeaux, Sylvia Pietri, Didier Morin, Yves Donazzolo, Jean-Louis Abitbol, Rebacca M Pruss, Sophie Schaller

**Affiliations:** 1 Trophos, Luminy Biotech Entreprises, Case 931, 13288 Marseille Cedex 9, France; 2 INSERM U955, Equipe 3, Université Paris Est, Faculté de Médecine, Créteil 94000, France; 3 Aix-Marseille Université, CNRS UMR 7273, Sondes Moléculaires en Biologie & Stress Oxydant, Institut de Chimie Radicalaire, Faculté des Sciences de Saint Jérôme, case 522, Avenue Escadrille Normandie-Niemen, 13397 Marseille Cedex 20, France; 4 Optimed Lyon, Pavillon 4 N 4O, Centre hospitalier Lyon sud 69 310, Pierre Bénite-France

**Keywords:** Acute myocardial infarction, Functional recovery, Mitochondrial permeability transition pore, Phase I clinical trial

## Abstract

**Background:**

Although reperfusion injury has been shown to be responsible for cardiomyocytes death after an acute myocardial infarction, there is currently no drug on the market that reduces this type of injury. TRO40303 is a new cardioprotective compound that was shown to inhibit the opening of the mitochondrial permeability transition pore and reduce infarct size after ischemia-reperfusion in a rat model of cardiac ischemia-reperfusion injury.

**Methods:**

In the rat model, the therapeutic window and the dose effect relationship were investigated in order to select the proper dose and design for clinical investigations. To evaluate post-ischemic functional recovery, TRO40303 was tested in a model of isolated rat heart. Additionally, TRO40303 was investigated in a Phase I randomized, double-blind, placebo controlled study to assess the safety, tolerability and pharmacokinetics of single intravenous ascending doses of the compound (0.5 to 13 mg/kg) in 72 healthy male, post-menopausal and hysterectomized female subjects at flow rates from 0.04 to 35 mL/min (EudraCT number: 2010-021453-39). This work was supported in part by the French Agence Nationale de la Recherche.

**Results:**

In the vivo model, TRO40303 reduced infarct size by 40% at 1 mg/kg and by 50% at 3 and 10 mg/kg given by intravenous bolus and was only active when administered before reperfusion. Additionally, TRO40303 provided functional recovery and reduced oxidative stress in the isolated rat heart model.

These results, together with pharmacokinetic based allometry to human and non-clinical toxicology data, were used to design the Phase I trial. All the tested doses and flow rates were well tolerated clinically. There were no serious adverse events reported. No relevant changes in vital signs, electrocardiogram parameters, laboratory tests or physical examinations were observed at any time in any dose group. Pharmacokinetics was linear up to 6 mg/kg and slightly ~1.5-fold, hyper-proportional from 6 to 13 mg/kg.

**Conclusions:**

These data demonstrated that TRO40303 can be safely administered by the intravenous route in humans at doses expected to be pharmacologically active. These results allowed evaluating the expected active dose in human at 6 mg/kg, used in a Phase II proof-of-concept study currently ongoing.

## Background

According to the World Health Organisation, cardiovascular diseases remain one of the leading causes of death in the world. A large number of people suffering from cardiac ischemia (e.g. myocardial infarction) die or suffer from cardiac dysfunction leading very often to a new cardiovascular event in the following years. These dismal statistics make it indisputable that these diseases are to be considered as having highly unmet medical needs.

During an acute myocardial infarction, ischemia and subsequent reperfusion each contribute to the loss of viable myocardium leading to alterations in left ventricular structure remodelling, reduced heart function and heart failure. Early myocardial reperfusion with the use of thrombolytic therapy or primary percutaneous coronary intervention (PCI) is the most effective strategy for reducing myocardial infarct size and improving the clinical outcome. However, because myocardial reperfusion paradoxically induces cardiomyocyte death [1,2], there has been an increased recognition of the need for treatments that reduce reperfusion injury [3-8]. Evidence indicates that opening of the mitochondrial permeability transition pore (mPTP) is largely involved in reperfusion injury [9] and that inhibitors of mPTP such as Cyclosporine A (INN: ciclosporin) can reduce reperfusion injury [10]. TRO40303 ( 3,5-seco-4-nor-cholestan-5-one oxime-3-ol) is a newly discovered compound that inhibits mPTP opening via an innovative mode of action [11]. TRO40303 is targeted to the cardiac mitochondria likely through binding to the cholesterol site of the outer mitochondrial membrane translocator protein (TSPO). TRO40303 has been shown to reduce infarct size in a model of myocardial infarction in rats while at the same time inhibiting AIF (Apoptosis Inducing Factor) release from mitochondria to the cytosol in the ischemic area, potentially via the reduction of oxidative stress [11]. However TRO40303 does not increase the calcium retention capacity of isolated mitochondria as does Cyclosporine A, probably because TRO40303 inhibits the mPTP by another mechanism than Cyclosporine A, which targets the mitochondrial matrix protein Cyclophilin D.

Translating a potential therapeutic from the bench to the bedside for an emergency indication such as myocardial infarction presents several important challenges. These include 1) establishing a pharmacokinetic-pharmacodynamic (PK/PD) relationship, 2) identifying the therapeutic window with respect to the clinical setting, 3) developing a formulation able to rapidly and safely deliver an effective dose and 4) confirming safety and PK parameters in healthy volunteers. This has not always been adequately investigated in the case of some previously developed cardioprotective compounds [12-14]. These issues are especially challenging with a poorly soluble or highly hydrophobic compound such as TRO40303 and for an acute care emergency indication such as an acute myocardial infarction where 1) time is critical so injection time is short making it important to find an excipient that maximizes solubility to minimize volume and injection time and 2) patients are unstable so less able to tolerate side effects known to be associated with emulsion formulations used to deliver poorly soluble compounds.

To address these challenges, we had to develop specific formulations for the preclinical and the clinical investigations. Previous studies showed that 2.5 mg/kg of TRO40303 [11], formulated in hydroxypropyl-beta-cyclodextrine (HPBCD) reduced infarct size by 38% in a myocardial infarction model in rats [11]. Because of the limited solubility in HPBCD, the maximum dose that could be tested using this vehicle was limited by the volume that could be administered (5 mL/kg). To increase exposure and explore a full dose-response relationship, TRO40303 was formulated in Intralipid® 30 (IL30), an oil-based parenteral nutritional supplement that provides improved TRO40303 solubility to concentrations up to ~6 mg/mL. Using this IL30 formulation, we evaluated the cardioprotective effects of TRO40303 by evaluating infarct size in a model of myocardial infarction in rats in terms of dose-effect relationship and therapeutic window in greater detail to derive a PK/PD relationship and to identify the optimal time of administration. Further studies also explored the functional recovery and gathered further evidence for TRO40303′s mechanism of action in an *ex vivo* model of cardiac ischemia-reperfusion injury. Recovery of left ventricular function, as assessed in the *ex vivo* model, is of extreme importance as together with infarct size, as assessed in the *in vivo* model, left ventricle dysfunction has been shown to be correlated with mortality following myocardial infarction in the clinic [15]. These data, together with pharmacokinetic allometric scaling to human, were used to estimate the expected active dose in humans allowing with the toxicological findings to design a dose escalating Phase I trial to investigate the safety, tolerability and pharmacokinetics of a single intravenous dose of TRO40303 in preparation for a Phase II clinical trial.

## Methods

### Reagents

All reagents were purchased from Sigma-Aldrich (St. Louis, MO) unless specified. TRO40303 [11] was synthesized by Synkem (Dijon, France). For *ex vivo* experiments, TRO40303 was dissolved in a solution of 30% HPBCD in phosphate buffered saline at 0.5 mg/mL (± 10%). In preclinical *in vivo* studies, TRO40303 was prepared as an emulsion in IL30, at ~6 mg/mL. Aqueous stock solutions of the nitrone 5,5-dimethyl-1-pyrroline *N*-oxide (DMPO) were purified and checked for diamagnetism as previously described [16]. For the clinical study, TRO40303 was administered as a 20 mg/mL solution in liposomes (Northern Lipids Inc, Burnaby, Canada).

### Animals

All animal procedures used were in strict accordance with the Directive 2010/63/EU of the European Parliament. Trophos, CNRS/Aix-Marseille University and INSERM/Institut Mondor de Recherche Biomédicale, had valid licences for animal experimentation (agreements B13-055-15, C13-055-06, C94-028-245) delivered by the French Government. Male Wistar rats (Janvier, Le Genest Saint Isle, France, 250–280 g) were used for *in vivo* myocardial infarction models. Male Sprague Dawley rats (SD, Harlan and CERJ, France, 250–300 g) were used for the *ex vivo* isolated heart and pharmacokinetic studies. Animals were maintained in the local animal house under conventional conditions, in a room with controlled temperature (21–25°C) and a reverse 12 h light/dark cycle with food and water available ad libitum.

### Human subjects

Men and post-menopausal or hysterectomized women aged from 18 to 70 years were eligible to participate in the Phase I trial with the following inclusion criteria: body mass index within 18-29 kg/m^2^ (inclusive), healthy as assessed by physical examination, medical history, vital signs, electrocardiography and all other clinical evaluations performed at screening and admission. All subjects gave written informed consent before any study-related procedures. The study was conducted at a single centre (Optimed Lyon, Lyon, France) after approval by the French Regulatory Agency AFSSAPS and the Ethics Committee (Comité de Protection des Personnes IV Lyon, France). The study was carried out in accordance with the Declaration of Helsinki (1964) as modified in Seoul (2008), the recommendations on Good Clinical Practice (ICH E6) and the applicable French regulatory requirement. The trial was registered with the EudraCT number: 2010-021453-39. All the data were collected and analysed at Optimed Lyon apart from the pharmacokinetic analysis.

### Myocardial infarction model

In all groups of rats, the left anterior descending coronary artery was occluded during 35 min and released for reperfusion as previously described [17]. For the dose range testing, the vehicle (IL30) or TRO40303 solution in IL30 (stock solution diluted in IL30 to administer ~1.5 mL/kg in each group) at increasing doses (0.3, 1, 3 and 10 mg/kg) were administered as a 5 min infusion through the jugular vein starting 10 min before coronary artery reperfusion (CAR). To evaluate the therapeutic window, the vehicle or TRO40303 at 1 mg/kg were also administered either 10 min before coronary artery occlusion (CAO) or 10 min after CAR. Infarct size was determined by triphenyltetrazolium chloride (TTC) staining and was calculated as a percentage of the area at risk (AAR), 24 h after reperfusion. For the dose range experiment, infarct size was expressed for all doses as a percent of the vehicle group infarct size.

### Pharmacokinetics and TRO40303 heart level measurement in rats

TRO40303 was administered by intravenous (i.v.) bolus to healthy rats at doses of 0.3, 1, 3 and 10 mg/kg in IL30 under isoflurane gas anaesthesia. A maximum of 0.25 mL of blood was collected into lithium heparin tubes at 5, 15 and 30 min, 1 h, 2 h, 4 h, 8 h and 24 h after drug administration (3 rats per time point). The blood samples were cooled on ice and plasma samples were prepared within 60 min of sampling by centrifugation at 1500 *g* at 4˚C for 10 min and stored at –20°C until analysis. For heart level measurements, TRO40303 was administrated i.v. to rats undergoing the myocardial infarction procedure at the dose of 1 mg/kg in IL30 either 10 min before CAO or 10 min before CAR. Five min after CAR, rats were anesthetised and sacrificed, hearts were isolated and washed with saline. Tissue from AAR was separated from the remainder of the left ventricle; samples were frozen and stored at –20°C until analysis. After TRO40303 plasma concentration analysis, TRO40303 plasma AUC_(0-∞)_ (the area under the curve extrapolated from time zero to time infinity), was calculated using Kinetica™, version 4.4.1 (Thermo Electron Corporation, Philadelphia, PA 19103, USA). The results were analyzed in comparison with previous pharmacokinetic data obtained with TRO40303 formulated in HPBCD at 2.5 mg/kg [11].

### Perfusion conditions in isolated heart model and hemodynamic measurements

Ischemic reperfusion of Langendorff isolated rat heart was performed as previously described [18]. Rat hearts were perfused either with TRO40303 1 μM (0.5 mg/mL stock in 30% HPBCD diluted 1000X in Krebs-Henseleit (KH) buffer (n = 29)) or vehicle (30% HPBCD diluted 1000X in KH buffer (n = 24)) during 30 min equilibration, 10 min low flow ischemia, and 60 min reperfusion following 30 min of total ischemia. During the reperfusion period, the following parameters were monitored via a water-filled latex balloon inserted into the left ventricle: left ventricular end diastolic pressure (LVEDP); left ventricular developed pressure (Pdev); its first derivative (dP/dt) and heart rate. Coronary flow was measured by collecting the coronary perfusate over 1 min. The rate-pressure product (RPP) was calculated as the product of Pdev and heart rate. Some of the hearts from this main experiment were used to assess ascorbate, reactive oxygen species (ROS) and lipid peroxidation as described below.

### Free radical and lipid peroxidation measurements

To evaluate the effect of 1 μM TRO40303 on ischemia-reperfusion-induced ascorbate release and tissue depletion, effluents from control and treated hearts undergoing the perfusion protocol described above (n = 6/group) were collected for 15 s, 1 min following onset of reflow, and hearts were frozen in liquid nitrogen at the end of the reperfusion period. Ascorbate content in effluents and tissue homogenates was then assessed by quantifying the ascorbyl free radical (AFR)-dimethyl sulfoxide (DMSO) electron spin resonance (ESR) doublet as previously described [19], DMSO being used to stabilize the ascorbate free radical.

To investigate whether TRO40303 could interfere with ischemia-reperfusion-triggered ROS production, hearts (n = 6) undergoing the perfusion protocol described above received the spin trap nitrone DMPO (25 mM) during the first 5 min of reperfusion. A 15-s collection of the effluents was performed pre-ischemically on three additional hearts from both groups, and 3 min after onset of reflow for the 6 ischemic/reperfused hearts. Samples were immediately stored in liquid nitrogen. The free radical content of DMPO-supplemented from TRO40303-treated and control effluents was determined by ESR in glass capillaries. The ESR signal was acquired 1.5 min after thawing of the sample using the same parameters as for ascorbate [19] except: modulation amplitude 0.0313 mT, receiver gain 5 × 10^4^ and sweep rate for each scan 0.167 mT/s.

Lipid peroxidation in heart tissue at the end of reperfusion was evaluated using the malondialdehyde-thiobarbituric acid assay (MDA-TBA) (n = 8/group) as previously described [18].

### Complement reactogenicity in normal human sera

Reactogenicity of the liposomal formulation of TRO40303 at a final concentration of 5.5 mg/mL was assayed *in vitro* for complement activation ability. SC5b-9 levels were measured by a commercial enzyme-linked immunosorbent assay (ELISA, Quidel Corporation; SC5b-9 Plus EIA kit, Ref. A029), following the instructions of the provider, on individual serum samples obtained from 10 healthy volunteers at Seroscience, Hungary. Blood withdrawal and experimentation have been carried out under conditions approved by the regional research ethics committee (Regional and Institutional Committee of Science and Research Ethics, Semmelweis University. Registration number: TUKEB 142/2008). AmBisome®, a liposome-containing antifungal drug, amphotericin B, was used at 4 mg/mL for final lipid concentration as a positive control. Data were compared to basal levels with no product added to the sera.

### Porcine liposome-induced cardio-pulmonary distress model

Domestic Yorkshire pigs (12-14 weeks, ~25 kg) received multiple i.v. injections of the test material via the external jugular vein at 10 mL/min as follows: saline as a negative control, the liposomal formulation of TRO40303 at 10 mg/kg and a bolus of Doxil® at 0.01 mg/kg as a positive control. Doxil® is a marketed liposomal formulation of doxorubicin administered intravenously that can provoke acute infusion-related reactions [20].

Hemodynamic changes were monitored continuously for at least 30 min after the end of each test material infusion. Systemic arterial blood pressure (SAP) and pulmonary arterial blood pressure (PAP) changes were evaluated.

### Pharmacokinetic based allometry

Pharmacokinetic based allometry (natural logarithm (ln) transformation) was performed using available pharmacokinetic data (not shown) from various animal species and was used to estimate clearance (CL) and the initial volume of distribution (V_1_ = Dose/C_0;_ C_0_ defined as the extrapolated plasma concentration at time zero) in human. Animals had received a single i.v. bolus dose of TRO40303 administered under lipid-based formulations (Intralipid® and liposomes) in rats and pigs or under cremophor EL®/ethanol/saline formulation in dogs and monkeys. The pharmacokinetic parameters were assessed from the mean (for rodents) or individual TRO40303 plasma concentration versus time profiles using a non-compartmental approach “Log Linear Method” (Kinetica™, version 4.4.1). The ln of the CL and the ln of V1 were each plotted against the ln of the animals’ body weight. Finally, using this relationship, the human CL and V_1_ were estimated based on the human body weight ranging from 50 to 115 kg.

### Clinical trial design

A randomized, double-blind, placebo controlled study was performed to investigate the safety, tolerability and pharmacokinetics of single i.v. ascending doses of TRO40303 in healthy male and post-menopausal female subjects. The choice of the dose and flow rate escalation process is explained in the Result section. Seventy-two healthy volunteers were randomized, with 18 receiving a placebo and 54 an active liposomal formulation containing 20 mg/mL TRO40303 at final doses ranging from 0.5, 1, 2, 3.5, 6, 10 to 13 mg/kg with escalating flow rates from 0.04 mL/min to 35 mL/min. Within each group, 3 or 6 subjects received TRO40303 and 1 or 2 subjects received the same volume of placebo. The sample size was based on the number of doses and flow rates to be investigated. The 6 verum/2 placebo ratio per group is widely used in single ascending dose studies and provides adequate safety and pharmacokinetic information. The 3 verum/1 placebo ratio used in the last groups was chosen only to explore additional flow rates at previously studied doses.

The study started on the 14^th^ of September 2010 and was completed on the 24^th^ of February 2011. The demographic characteristics of the recruited subjects are presented in Additional file 1: Table S1. The randomization list was produced by a statistician from Optimed Lyon and established by dose and flow rate. Subjects were assigned a chronological treatment number from 001 to 072. All participants from Trophos and Optimed Lyon as well as subjects were blinded to the study, except from the pharmacist at Optimed Lyon. The blinding was achieved by using aluminum paper to cover the content of the syringes and tubes. Additionally, the analysis of TRO40303 plasma level at Bertin Pharma was performed un-blinded.

For each dose group, a decision to proceed to the next higher dose was made based on blind safety/tolerability data on at least 75% of the subjects up to at least 48 h post-dose. Pharmacokinetic data up to 24 h was available at the time of decision.

### Sampling scheme and Pharmacokinetic parameters

Venous blood samples (5 mL) were collected for determination of TRO40303 concentrations in plasma at pre-dose, end of infusion, then 5 min, 15 min, 30 min, 1 h, 2 h, 4 h, 8 h, 12 h, 16 h, 24 h, 48 h, 72 h and 96 h post end of infusion into heparin–lithium Vacutainer tubes. The blood samples were gently inverted then centrifuged in a refrigerated centrifuge (+4°C) within 30 min after sampling at 1500 *g* for *ca* 10 min. The resulting plasma was frozen in two separate labelled polypropylene tubes at –20 ± 5°C until assaying.

After TRO40303 plasma concentration analysis, the following parameters were calculated for each subject: TRO40303 maximum plasma concentration (C_max_), TRO40303 plasma concentration at 5 min post end of infusion (C_5min_), AUC_(0-∞)_, plasma elimination half life (t_half_), CL and volume of distribution (V_z_), using Kinetica™, version 4.4.1 at Trophos.

### Quantification of TRO40303 in plasma and heart samples by HPLC-MS/MS

Thawed preclinical samples were extracted with acetonitrile, centrifuged, purified on SPEC C2 cartridge (Varian) for the heart samples and analysed along with calibration standards. Analysis was carried out on an Alliance 2695 (Waters) system interfaced to an API Quattro Micro (Waters) MS detector. Calculations were carried out with Waters QuanLynx software.

For the quantifying TRO40303 levels in human plasma samples, the same method, GLP validated, was used and the analysis was performed at Bertin Pharma (Orleans, France). Analysis was carried out on 1200 Series (Agilent) and Series 200 (Perkin Elmer) systems interfaced to an API 4000 (AB Sciex) MS detector. Calculations were carried out with Analyst 1.4.1 and 1.5.1 (AB Sciex) software.

### Safety and tolerability

Continuous recording of adverse events, continuous blood pressure monitoring for 2 hours after dosing and 12-lead electrocardiogram (ECG) at pre-dose, 15 min, 1 h, 4 h, 8 h, 24 h, 72 h and 96 h after end of infusion were performed. Vital signs, physical examination, haematology, blood biochemistry, urinalysis and haemostasis were performed before the study, at specific times after dosing and at the “end of study visit”, 7 days after administration. Blood biochemistry analysis included ASAT, ALAT, alkaline phosphatase, triglycerides, total cholesterol, HDL-cholesterol and LDL-cholesterol.

To assess any risk of complement activation related pseudo-allergy (CARPA) reaction in the clinical study, SC5b-9 levels were measured by a commercial enzyme-linked immunosorbent assay (ELISA, Quidel Corporation; SC5b-9 Plus EIA kit, Ref. A029), following the instructions of the provider at Seroscience, Hungary. Serum samples for SC5b-9 analysis were collected as described for TRO40303 PK analysis before dosing, and then 5, 15 and 60 min after end of infusion.

### Statistical data analysis

The preclinical data are reported as mean ± S.E.M. Statistical significance was determined using either two-sided Student’s t-test or one-way analysis of variance (ANOVA) followed by Dunnett’s post test. Significance was accepted when p < 0.05. The exact p value is given when 0.01 < p < 0.05.

For the clinical data, descriptive statistics were used. All randomized subjects were included in the analysis.

## Results

### Dose-effect relationship of TRO40303 in an *in vivo* rat model

**Figure 1:**
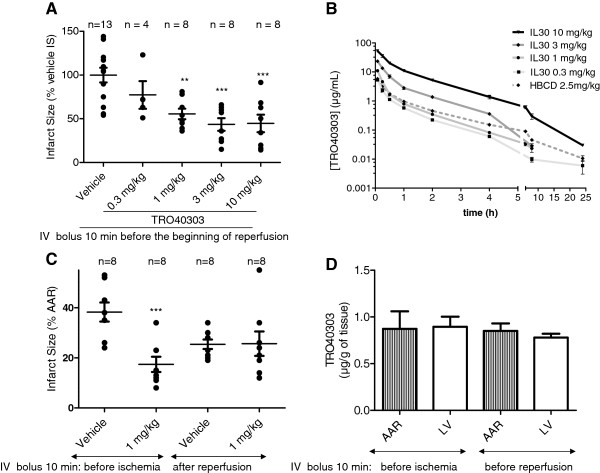
**Dose effect and therapeutic window of TRO40303 in a myocardial infarction model in rats.** Infarct sizes (IS) were quantified after 24 h coronary artery reperfusion (CAR) following 35 min of coronary artery occlusion (CAO), and calculated for each group as percentage of the area at risk (AAR) and then expressed as a percentage of the IS of the control group for each dose. N is the number of treated rats per group; circles are individual values while the mean ± S.E.M is indicated by black bars for each group; **: p < 0.01 and ***: p < 0.001 versus control. **A)** TRO40303 formulated in IL30 was administrated by i.v. bolus to rats 10 min before CAR. The doses of 1, 3 and 10 mg/kg provided significant protection in this ischemia-reperfusion model while 0.3 mg/kg did not have a significant effect using ANOVA followed by Dunnett’s post test. The average IS of the control group (vehicle 100%) corresponded to 43.62 ± 0.04% of the AAR. **B)** Plasma level of TRO40303 expressed in μg/mL during 24 h after i.v. bolus to satellite rats corresponding to the four doses tested in the IL30 formulation and compared to a pharmacokinetic profile of the 2.5 mg/kg dose in HPBCD previously shown as active. **C)** One mg/kg TRO40303 formulated in IL30 reduced infarct size significantly when administrated by i.v. bolus to rats 10 min before CAO, but not 10 min after CAR as evaluated by Student T-Test. **D)** Heart level of TRO40303 at 5 min after reperfusion in ischemic (AAR) or remainder of left ventricle (LV) after administration of 1 mg/kg TRO40303 formulated in IL30.

Using the IL30 formulation, doses ranging from 0.3 to 10 mg/kg were investigated in a model of myocardial infarction in rats. TRO40303 or IL30 vehicle alone were administered by i.v. bolus in a constant volume 10 min before reperfusion following a 35 min CAO. Whereas infarct size determined 24 h after reperfusion was smaller but not significantly different at the lowest tested dose of 0.3 mg/kg, 1 mg/kg significantly reduced infarct size by 40% (p < 0.01) as compared to vehicle–treatment. The effect of TRO40303 reached a plateau of ~50% reduction of infarct size compared to control (p < 0.001) with doses of 3 and 10 mg/kg (Figure 1A and Table 1).

**Table 1:** Effects of TRO40303 on infarct size after cardiac ischemia reperfusion in rats

**Dose (mg/kg)**	**AAR (% LV)**		**IS (% LV)**		**IS (% AAR)**		**IS (% Vehicle IS)**	
	**TRO40303**	**Vehicle**	**TRO40303**	**Vehicle**	**TRO40303**	**Vehicle**	**TRO40303**	**Vehicle**
**0.3**	45 ± 5	54 ± 4	11 ± 2	18 ± 3	26 ± 4	33 ± 4	77 ± 15	100 ± 13
**1**	39 ± 4		7 ± 1		19 ± 2		56 ± 6	
**3**	43 ± 4		6 ± 1		15 ± 2		44 ± 7	
**10**	30 ± 2	35 ± 3	7 ± 2	20 ± 3	25 ± 6	56 ± 6	45 ± 10	100 ± 10

For pharmacokinetic studies in healthy rats, TRO40303 was administered formulated in IL30 at the doses used in the myocardial infarction model and results analyzed in comparison with pharmacokinetic data obtained with TRO40303 formulated in HPBCD at 2.5 mg/kg. When TRO40303 was formulated in IL30, exposure was higher than previously found with TRO40303 formulated in HPBCD. The dose of 1 mg/kg TRO40303 in IL30 provided a similar exposure as that obtained with 2.5 mg/kg in HPBCD (Figure 1B). This correlated well with the level of activity found with both doses in the two formulations.

The TRO40303 plasma exposure in rats in terms of C_5min_ and AUC_(0-∞)_ together with the corresponding activity in term of infarct size reduction for each dose, were used to establish a PK/PD relationship.

The plateau of activity corresponding to ~50% infarct size reduction was determined as the maximum achievable cardioprotective activity for TRO40303 in this model and doses achieving this level of infarct size reduction thus corresponded to the target plasma exposure to achieve. The pharmacokinetic parameters for the fully active doses of 3 and 10 mg/kg in rats, measured in satellite animals similarly infused, were: C_5min_, 57.9 and 130 μM (23.5 and 52.8 μg/mL) and AUC_(0-∞)_ of 14.0 and 44.8 μg.h/mL, respectively. The exposure corresponding to the dose of 3 mg/kg was defined as the minimum fully effective plasma exposure.

### Therapeutic window of TRO40303

The therapeutic window for TRO40303 treatment was investigated in rats by administering the lowest active dose of 1 mg/kg in IL30 either 10 min prior to 35 min CAO or 10 min after the onset of CAR. The results showed that when administered before CAO 1 mg/kg TRO40303 reduced infarct size by 55% compared to vehicle (versus 40% when administered 10 min prior to reperfusion), whereas infarct size was unchanged compared to vehicle–treated rats when TRO40303 was administered 10 min after the onset of reperfusion (Figure 1C). However, in this case it must be noted that the extent of the infarct was lower in the vehicle group when treated 10 min after CAR compared to administration before CAO (Figure 1C). Nevertheless, TRO40303 provided no greater effect when administered 10 min post reperfusion. Thus TRO40303 should be administered before the beginning of reperfusion in order to provide cardioprotection.

The concentration of TRO40303 in heart tissue was measured both in the ischemic AAR and in the remainder of the left ventricle. These data showed that when given prior to ischemia, TRO40303 remains in the heart during the ischemic period and is detectable at a similar level 5 min post reperfusion as that found when the compound was administered 10 min prior to reperfusion (~1 μg/g; Figure 1D), which shows that TRO40303 is rapidly accumulated into myocardial tissue and remains stable for at least 1 h.

### Functional recovery and reduction of oxidative stress by TRO40303

In order to assess if the reduction of infarct size by TRO40303 treatment in the *in vivo* myocardial infarction model translates into meaningful functional recovery of the heart, an *ex vivo* model of ischemia-reperfusion using the Langendorff isolated rat heart was used to measure heart function by monitoring the pressure of the left ventricle during ischemia and reperfusion.

**Figure 2:**
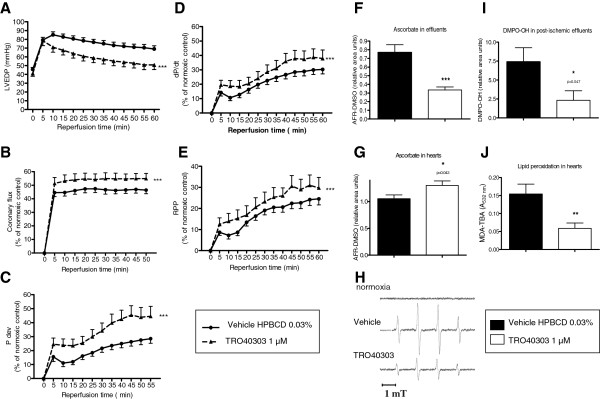
**Functional recovery and reduction of oxidative stress by TRO40303 in isolated rat hearts following ischemia-reperfusion.** Isolated rat hearts were perfused using the Langendorff system by solutions of KH buffer containing 0.03% HPBCD with or without TRO40303 1 μM. **A**: left ventricular end diastolic pressure (LVEDP), **B**: coronary flow, **C**: developed pressure (Pdev), **D**: contractility (dP/dt) and **E**: rate pressure product (RPP) was evaluated in isolated rat hearts during 60 min of reperfusion following 30 min of no flow ischemia. **A to E**: Comparisons among groups were performed using a two-way ANOVA, ***: p < 0.001 vs 0.03% HPBCD. A significant improvement in functional recovery was observed for all the parameters when hearts were perfused with KH buffer containing TRO40303. Relative ascorbate levels were measured through ESR detection of dimethyl sulfoxide (DMSO)-stabilized ascorbyl free radical (AFR) in the effluents **(F)** at the 1^st^ min of reperfusion and in the heart tissue **(G)** at the end of reperfusion. TRO40303 reduced the level of ascorbate release in the effluents while maintaining a higher level in heart tissue. DMPO-OH release was evaluated in the coronary effluents from hearts reperfused in the presence of 25 mM DMPO: typical spectra **(H)** recorded 3 min following onset of reflow for vehicle and TRO40303-treated samples; no signal was observed when hearts were perfused with 25 mM DMPO during normoxia; and determination of relative DMPO-OH levels **(I)** showing that TRO40303 1 μM reduced post-ischemic ROS release. Lipid peroxidation, evaluated by MDA-TBA in the heart ventricle at the end of reperfusion, was reduced in the hearts treated by TRO40303 compared to vehicle **(J)**. **F to J**: Bars indicate mean ± S.E.M. and comparisons among groups were performed using a t-test *: p < 0.05, **: p < 0.01 vs 0.03% HPBCD (n = 6/group).

As seen by the monitoring of LVEDP (Figure 2A), the contracture induced by the ischemic period was attenuated at the time of reperfusion. The extent of the recovery was greatly and significantly improved in hearts exposed to 1 μM TRO40303. This was accompanied by better recovery of coronary flow (Figure 2B), thus allowing an improved Pdev (Figure 2C) and contractility (dP/dt, Figure 2D). The improvement of global mechanical performance in TRO40303–treated hearts was evaluated at reperfusion by calculating the RPP, which was significantly higher (Figure 2E).

It has been shown that at least part of TRO40303′s mechanism of action, that correlated with cardiomyocyte protection *in vitro,* involves a reduction in oxidative stress and ROS production [11]. We used the isolated heart model to investigate if the same mode of protection could be observed in the full organ. To address this question, in a first set of experiments, the levels of AFR-DMSO, which relates to ascorbate free radical content [19] were measured by ESR spectrometry in post-ischemic coronary effluents and myocardial tissue in control and TRO40303–treated isolated hearts. It was previously reported that an early post-ischemic effluent release concomitant to a decrease in tissue AFR-DMSO was detected in untreated rat hearts during the reperfusion phase [19]. Supplementing the perfusion medium with 1 μM TRO40303 significantly inhibited AFR-DMSO release in the coronary effluents during early reperfusion (–57% versus untreated hearts, p < 0.001 (n = 6); Figure 2F) and prevented tissue AFR-DMSO depletion at the end of reflow (+24% versus untreated hearts, p < 0.05 (n = 6); Figure 2G).

In a second set of experiments, the effect of infusing TRO40303 on spin adduct formation in hearts reperfused in the presence of the nitrone DMPO was investigated. In both groups of hearts when the spin trap (25 mM) was added to KH medium during the last minute preceding ischemia, no ESR signal was detected in effluent samples showing that ROS production was below the level of detection during normoxia (Figure 2H). DMPO and other structural analogues have been shown to inhibit post-ischemic spin adduct formation by protecting the heart during ischemia [21,22]. In order to minimize this potential cardioprotection by DMPO, two additional groups of vehicle and TRO40303–treated hearts received the spin trap (25 mM) only at reperfusion and the spin adduct content of coronary effluents was determined 3 min after the onset of reflow, a time when maximal spin adduct production has been reported [16,23]. In both groups of hearts a 1:2:2:1 ESR quartet characteristic of DMPO-OH, the hydroxyl radical (HO) DMPO adduct, was detected; however, the intensity of this signal was decreased by 69% in the TRO40303–treated group (p < 0.05 (n = 6); Figure 2H and I).

In a third set of experiments, MDA-TBA was used as an index of myocardial lipid peroxidation. Figure 2J shows that pre-treatment of hearts with 1 μM TRO40303 significantly limited MDA-TBA formation as compared to vehicle-perfused hearts (–65%, p < 0.01 (n = 8)).

### Dose selection for Phase I trial

The doses used in the Phase I trial were based on 1) the starting dose calculated with the toxicology findings and 2) the predicted active dose in human that was calculated based on two points: the target TRO40303 plasma exposure (C_5min_, AUC_(0-∞)_) that correlated with the minimum fully effective dose resulting in 50% reduction of infarct size in rats; and the predicted pharmacokinetic parameters in human that were calculated by allometric scaling.

The starting dose of 0.5 mg/kg was calculated from FDA guidelines [24] based on findings from non-clinical toxicology studies (not shown).

**Figure 3:**
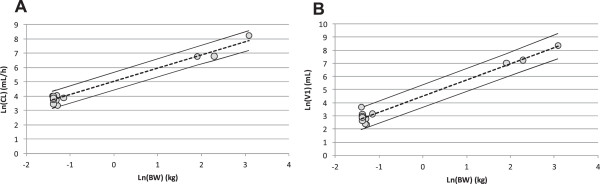
**Allometry scaling to human.** Clearance CL **(A)** and the initial volume of distribution V_1_**(B)** were plotted against the body weight (BW) of the animals (expressed in ln) allowing the estimation of the human CL and V_1_ based on the human BW as described in materiel and method section.

The CL, V_1_ and body weights of various preclinical species exposed to TRO40303 were used to extrapolate and predict human CL or V_1_ based on the relationship between the ln of these parameters with the inclusion of a 95% confidence interval (CI) (Figure 3A and B). From this allometric scaling, the predicted human CL and V_1_ estimated for body weights ranging from 50-115 kg (as expected in a future Phase II clinical trial), were 53-238 mL/h/kg and 89-741 mL/kg, respectively.

The active dose predicted for humans in this weight range was then calculated using the formulas, Dose = CL × AUC_(0-∞)_ or Dose = V_1_ × C_0_, considering that C_5min_ value was close to C_0_ value. Including a 95% CI, a human dose predicted to provide ~50% reduction in infarct size should be between 0.7 and 3.3 mg/kg based on CL or between 2.1 and 17 mg/kg based on V_1._ Therefore, the Phase I study was designed to cover doses ranging from 0.7-17 mg/kg.

### Clinical TRO40303 formulation development

**Figure 4:**
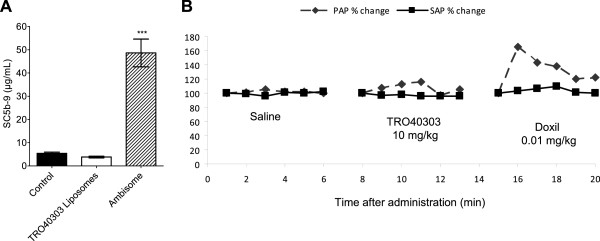
**Absence of CARPA reactions using the liposomal TRO40303 formulation. A)** Serum from 10 healthy volunteers were incubated with either TRO40303 liposomal formulation at 5.5 mg/mL or AmBisome® at 4 mg/mL. After 45 min at 37°C, detection of SC5b-9 was performed by ELISA and the mean values were compared with basal level of the sera with no compound addition. The TRO40303 liposomal formulation did not induce SC5b -9 release whereas AmBisome® induce a significant release (***: p < 0.001 versus control). **B)** Systemic Arterial blood Pressure (SAP) and Pulmonary Arterial blood Pressure (PAP) changes were evaluated in a male pig after i.v. injection of the same volume of either saline or 10 mg/kg TRO40303 liposomal formulation infusion at 10 mg/mL followed by a bolus of Doxil® at 0.1 mg/kg. Doxil® induced an increase in PAP whereas TRO40303 had no effect. The data presented here are typical of 5 independent experiments.

In order to be able to deliver an effective dose in a volume that can be administered in no more than a few minutes, a clinical formulation was needed that provided increased drug load over that of IL30. Because TRO40303 is highly hydrophobic but liposoluble, a clinical formulation in liposomes was developed having a concentration of ~20 mg/mL. However, liposomal preparations can induce hypersensitivity reactions [25,26] due to CARPA, which is related to the type of liposomes and the speed of injection [27]. To avoid this side effect, a TRO40303 liposomal formulation was developed and selected based on the failure to stimulate production of complement fragment, SC5b-9 in human sera. To this end, candidate liposomal formulations of TRO40303 were assayed *in vitro* and selected based on the failure to induce formation of SC5b -9 in a battery of human sera. The positive control in these studies was AmBisome®, a product that is known to induce CARPA reactions [28], which induced a large release (Figure 4A).

The selected formulation was then evaluated in an *in vivo* model of liposome-induced cardio-pulmonary distress in pigs, the most sensitive species for CARPA reactions [29]. Measuring PAP in this model has been shown to be a good indicator for human hypersensitivity to liposomal drugs [27]. I.v. bolus administration of liposomal TRO40303 at 10 mg/kg did not induce a significant change in PAP whereas Doxil®, a product with known susceptibility to induce infusion-related reactions [20], greatly increased it (Figure 4B). SAP was not changed either by TRO40303 or Doxil®.

### Design and results of the Phase I trial

**Figure 5:**
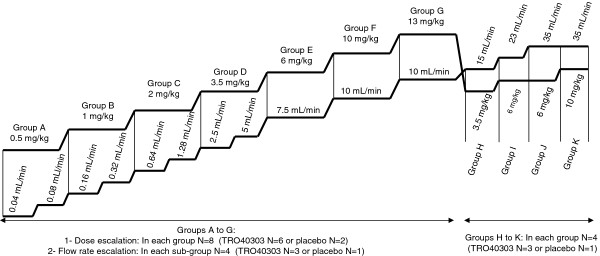
**Design of the Phase I clinical trial.** Sequential dose and flow rate escalation is presented per group of subjects in the Phase I trial. For the dose escalation, in each group A to G, consisting of eight subjects, six received TRO40303 and two the placebo; flow rate escalation was performed in sub-groups of four subjects. In groups H to K consisting of four subjects, three subjects received TRO40303 and one the placebo.

As the Phase I trial design was focused on evaluating the safety as well as PK parameters of TRO40303 administered intravenously, the level of SC5b-9 was carefully monitored and both the dose and infusion flow rate were escalated slowly as illustrated in Figure 5. The administration was initiated at a flow rate of 0.04 mL/min and aimed at achieving the intended flow rate of 10 mL/min. By the time the dose of 13 mg/kg at a flow rate of 10 mL/min was achieved, exposure levels were already higher than predicted so the trial protocol was amended to repeat lower doses but further increase the flow rates to 35 mL/min.

In this study, all the tested doses and flow rates were well tolerated clinically. There were no serious adverse events reported. During the overall study period, 14/72 (19.4%) subjects reported the occurrence of 14 adverse events. Four of these occurred before product administration (3 episodes of headache and one episode of catheter site pain) and 10 occurred after initiating product administration (emergent). Among emergent adverse events, 4 were experienced after administration of placebo. The list of the adverse events, their intensity and their relationship to the study product is presented in Table 2.

**Table 2:** List of adverse events (AEs)

	**Administration**	**Description**	**Relationship**	**Intensity**
Non emergent AEs (before product initiation)	0.5 mg/kg; 0.04 mL/min	1 episode of headache	Non applicable	Moderate
	0.5 mg/kg; 0.08 mL/min	1 episode of headache	Non applicable	Moderate
	13 mg/kg; 10 mL/min	1 episode of headache	Non applicable	Moderate
		1 episode of catheter site pain		
Emergent AEs (after product initiation)	Placebo	2 episodes of headache	1 Unlikely, 1 Possible	Mild
		1 episode of myoclonus	Possible	
		1 episode of chest pain	Unlikely	
	0.5 mg/kg; 0.08 mL/min	2 episodes of back pain	Excluded	1 Moderate and 1 Mild
	2 mg/kg; 0.64 mL/min	1 episode of injection site haematoma	Excluded	Moderate
	3.5 mg/kg; 5 mL/min	1 episode of presyncope	Unlikely	Moderate
	6 mg/kg; 7.5 mL/min	1 episode of infusion site inflammation	Probable	Moderate
	6 mg/kg; 23 mL/min	1 episode of vision blurred	Possible	Moderate

No relevant changes in vital signs, ECG parameters, laboratory tests or physical examinations were observed at any time in any dose group. Nearly all measurements were within the laboratory range or assessed as normal by clinical assessment. The out-of-reference range values for biochemical, hematological, vital signs and ECG parameters were judged by the investigators of no clinical significance and no clinically relevant changes were observed.

**Figure 6:**
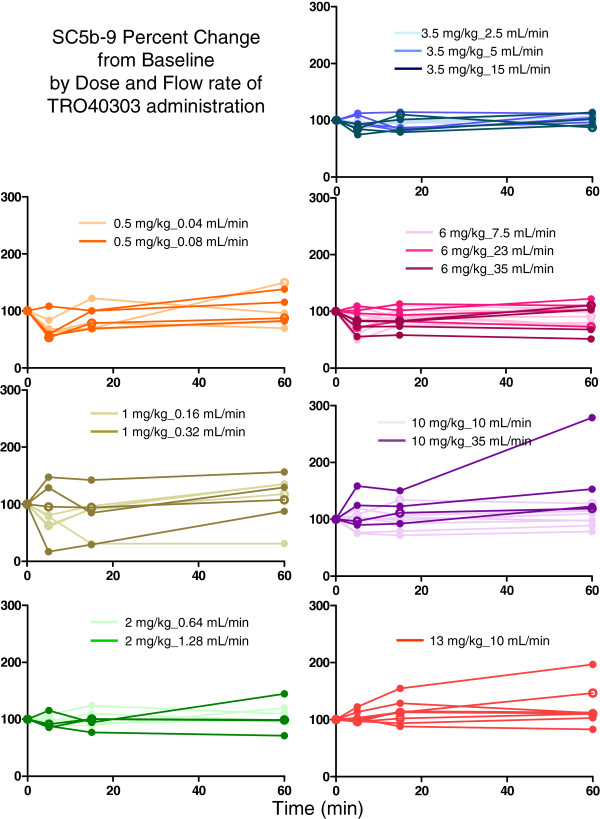
**Sc5b-9 plasma levels in human healthy volunteers of the Phase I trial.** Seventy-two healthy volunteers were administered increasing i.v. doses of either TRO40303 or placebo as described in the materiel and method section. Plasma level of SC5b-9 was dosed in all subjects before administration, and then 5, 15 and 60 min after end of infusion. Percent evolution from baseline is presented for each subject. Dose and flow rate groups are indicated by colour code. Plain circles represent subjects having received TRO40303 and open circles, subjects having received the placebo.

The evaluation of complement activation showed a slight biological activation for 4 subjects (one in the 13 mg/kg-10 mL/min group, one in the placebo group and two in the 10 mg/kg-35 mL/min group), none were judged clinically significant (Figure 6). This confirmed the safety of the TRO40303 liposomal formulation selected based on the failure to stimulate production of complement fragment SC5b-9.

**Figure 7:**
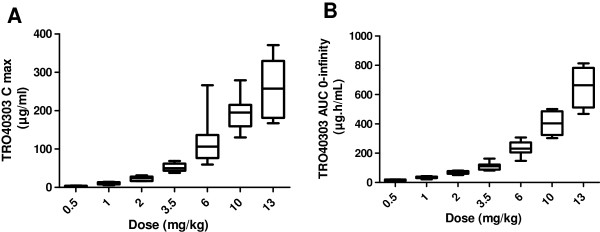
**TRO40303 plasma levels in human healthy volunteers of the Phase I trial.** TRO40303 plasma C_max_**(A)** and AUC_(0-∞)_**(B)** have been calculated for each exposed subject of the Phase I trial. The median as well as values for the lower and upper quartiles are presented by dose level as boxes.

TRO40303 pharmacokinetics in terms of C_max_ and AUC_(0-∞)_ were linear from 0.5 to 6 mg/kg and slightly ~1.5-fold hyper-proportional from 6 to 13 mg/kg (Figure 7 and Table 3). At 13 mg/kg, the mean C_max_ was 259 μg/mL and the mean AUC_(0-∞)_ was 651 μg.h/mL. The mean penultimate t_half_ (when at least 80% of drug was eliminated) was 4 h, mean terminal t_half_ was 25 h and V_z_ was 1.1 L/kg with a moderate inter-individual variability. Compared to the pharmacokinetic parameters in rats, C_5min_ is ~2-fold higher and AUC_(0-∞)_ is ~7-fold higher in humans for the same dose. These differences are probably in part due to the use of different formulations in the two species: IL30 in rats and liposomes in human and might also explain the difference between predicted CL and V_1_ parameters versus real data in human. The pharmacokinetic parameters per dose group and flow rate are presented in Table 3. These data were used to estimate the expected active dose to be used in the phase II clinical trial [30]. In order to maximize the potential dose in human, the highest plasma exposure leading to 50% reduction of infarct size as determined in the rat model to (C_5min_ = 52.8 μg/mL) was chosen as the target. The dose of 6 mg/kg should assure that all patients receive an effective dose based on C_5min_ which is the most relevant parameter for a product that should affect events occurring at the acute time of reperfusion.

**Table 3:** PK parameters for TRO40303 in healthy male and female subjects following single i.v. administration

**Parameter (unit)**		**Dose level (mg/kg)**						
		**0.5 (n = 6)**	**1 (n = 6)**	**2 (n = 6)**	**3.5 (n = 9)**	**6 (n = 12)**	**10 (n = 9)**	**13 (n = 6)**
**C**_**max **_**(μg/mL)**	Mean	3.32	10.3	23.1	51.7	118	194	259
	Min-max	2.31-4.85	6.06-14.1	16.0-31.1	37.7-68.7	59.5-266	130-279	167-371
	CV	28.5%	29.8%	25.4%	20.8%	47.3%	23.2%	29.7%
**C**_**5min **_**(μg/mL)**	Mean	2.58	9.26	23.6**	50.2	112	193	248
	Min-max	1.59-3.92	5.98-12.5	16.7-26.8	37.1-68.7	51.6-266	120-279	148-371
	CV	32.0%	26.7%	17.7%	22.9%	52.3%	24.3%	31.6%
**AUC**_**(0-∞) **_**(μg.h/mL)**	Mean	15.5*	34.5	68.6	109	229***	408	651
	Min-max	9.13-20.9	21.0-43.0	50.7-81.5	81.0-163	147-307	303-501	468-814
	CV	35.0%	21.8%	17.1%	23.6%	21.8%	18.8%	21.4%
**Terminal t**_**1/2 **_**(h)**	Mean	29.5	25.1	26.4	25.1	24.4	25.3	24.4
	Min-max	27.0-31.5	22.9-26.8	24.3-28.7	23.1-27.7	21.2-30.0	19.3-30.2	20.1-28.6
	CV	6.3%	5.6%	6.2%	7.0%	11.1%	12.8%	12.4%
**CL (mL/min/kg)**	Mean	0.598	0.509	0.499	0.560	0.460	0.422	0.347
	Min-max	0.399-0.912	0.388-0.792	0.409-0.657	0.357-0.721	0.326-0.680	0.332-0.549	0.266-0.463
	CV	39.3%	28.6%	18.7%	20.9%	25.3%	20.0%	22.7%
**V**_**z **_**(mL/kg)**	Mean	1.54	1.11	1.14	1.22	0.964	0.929	0.723
	Min-max	1.03-2.49	0.899-1.77	0.910-1.52	0.723-1.52	0.611-1.34	0.624-1.44	0.599-0.989
	CV	43.4%	30.5%	19.7%	20.5%	23.5%	26.8%	20.0%

*n = 4; **n = 5; ***n = 11.

## Discussion

These studies describe the successful translation of a new cardioprotective therapy, TRO40303 from preclinical proof of concept to clinical evaluation of safety, tolerance and measurements of human pharmacokinetic parameters. The results support the further development of TRO40303 to treat reperfusion injury in patients treated by PCI for an acute myocardial infarction. Preclinical ischemia-reperfusion models confirmed that TRO40303 dose-dependently preserved heart tissue and restored heart function. These studies also provided further evidence that TRO40303 reduces oxidative stress triggered by reperfusion, which along with calcium overload, triggers mitochondrial permeabilization underlying cardiomyocyte cell death [31].

Similar cardioprotective effects were found when TRO40303 was administered to rats either 10 min prior to 35 min left ventricular ischemia or 10 min before reperfusion but not when administered 10 min after the onset of reperfusion. This results show that TRO40303 targets early events, such as mitochondria permeabilization, leading to reperfusion injury but does not act on later reperfusion events. In the clinical setting, this has an important consequence: TRO40303 should be administered before reperfusion is performed either by angioplasty or thrombolysis. TRO40303 could thus be effective when administered in an emergency situation just before PCI or prophylactically in case of scheduled PCI or coronary artery bypass graft surgery.

The safety and tolerability of TRO40303 administered by i.v. infusion in humans was then verified in a Phase I trial. Intravenous doses of TRO40303 from 0.5 mg/kg to 13 mg/kg were all well tolerated. Infusion flow rates up to a maximum of 35 mL/min were tested with doses between 3.5 and 10 mg/kg and a dose of 6 mg/kg was predicted to be able to deliver a clinically effective level of TRO40303 at the time of reperfusion. Thus, it should be possible to deliver this dose in less than 1 min with TRO40303 formulated in liposomes at a concentration of 20 mg/mL, the desired objective for an emergency indication such as an acute myocardial infarction. The dose of 6 mg/kg assures that all individuals will achieve a C_5min_ above 52.8 μg/mL, the plasma exposure found in rats treated with 10 mg/kg that resulted in ~50% reduction in infarct size. At a lower dose it is possible that not all the individuals will have that level of exposure at the time of reperfusion based on the observed inter-individual variability. This dose is currently being evaluated in a Phase II clinical trial (ClinicalTrials.gov Identifier: NCT01374321) [30]. Notably, safety, tolerance and most importantly the lack of CARPA-type reactogenicity with TRO40303 formulated in liposomes, which is of particular importance for the security of patients being treated for an acute myocardial infarction, was confirmed in the Phase I study.

Limitations of these studies are that TRO40303 efficacy studies have only been performed in rodent models and that the formulation used in animal models is different from the formulation used in humans. Additionally, human safety and pharmacokinetics was only tested in 54 individuals exposed to TRO40403 and only 27 with doses of 6 mg/kg or higher. Further development of the product in patients is thus required in order to validate if similar safety, pharmacokinetics and efficacy is observed in the target population. Of note, it must be pointed out that several very promising therapies in preclinical models for cardiac reperfusion injury have failed in human.

## Conclusions

In conclusion, TRO40303 timing of administration, dose-dependent efficacy and functional effects were characterised in preclinical models of cardiac ischemia-reperfusion injury. Safety was demonstrated in humans in a Phase I trial and the pharmacokinetic parameters in humans were determined confirming the therapeutic potential of TRO40303 and the feasibility to perform a Phase II proof of concept trial, which is ongoing. The outcome of the Phase II trial will determine whether TRO40303 reduces infarct size in humans, and provide a path to developing it as a therapy to reduce reperfusion injury, cardiac remodelling and eventually morbidity and mortality following acute myocardial infarction.

## Abbreviations

AAR: Area at risk; AFR: Ascorbyl free radical; AEs: Adverse events; ANOVA: Analysis of variance; AUC(0-∞): Area under the curve extrapolated from time zero to time infinity; BW: Body weight; C5min: TRO40303 plasma concentration at 5 min post end of infusion; Cmax: TRO40303 maximum plasma concentration; CAO: Coronary artery occlusion; CAR: Coronary artery reperfusion; CARPA: Complement activated related pseudo-allergy; CI: Confidence interval; CL: Clearance; DMPO: 5,5-dimethyl-1-pyrroline *N*-oxide; DMSO: Dimethyl sulfoxide; ECG: Electrocardiogram; ESR: Electron spin resonance; HPBCD: Hydroxypropyl-beta-cyclodextrine; IL30: Intralipid® 30; i.v.: Intravenous; KH: Krebs-Henseleit; ln: Natural logarithm; LVEDP: Left ventricular end diastolic pressure; MDA-TBA: Malondialdehyde-thiobarbituric acid assay; mPTP: Mitochondrial permeability transition pore; PAP: Pulmonary arterial blood pressure; PCI: Percutaneous coronary intervention; Pdev: left ventricular developed pressure; ROS: Reactive oxygen species; RPP: Rate-pressure product; SAP: Systemic arterial blood pressure; thalf: plasma elimination half life; TRO40303: 3,5-seco-4-nor-cholestan-5-one oxime-3-ol; TTC: Triphenyltetrazolium chloride; V1: initial volume of distribution; Vz: Volume of distribution.

## Competing interests

AB has served as consultant and received honorarium from TROPHOS. SLL, CC, MM, PB, RMP JLA and SS are employees of TROPHOS and CC, MM, PB and RMP hold shares in TROPHOS.

## Authors’ contributions

SLL performed the pharmacokinetic analysis of the clinical trial and the allometry study, S.Pa. performed the *in vivo* rat myocardial infarction model experiments, HR performed the isolated rat heart experiments, CC was in charge of the human TRO40303 formulation development and the *in vitro* CARPA study, MM supervised the pharmacokinetic studies in preclinical models and was in charge of the CARPA *in vivo* study; MC was in charge of the nitrone/spin trap experiments, J.A. supervised all the analytical studies, ML supervised the phase I clinical trial, P.B. supervised chemical development and toxicology studies, AB designed the work on the *in vivo* rat myocardial infarction model experiments and revised the manuscript, RMP supervised all the preclinical and clinical development activities, contributed to study design and analysis and revised the manuscript, S.Pi designed the work on isolated rat heart experiments and revised the corresponding part of the manuscript, DM supervised the work on the *in vivo* rat myocardial infarction model experiments and revised the manuscript, YD was the investigator responsible for the phase I clinical trial and was involved in the design of the trial, JLA was the medical sponsor responsible for the phase I clinical trial and was involved in the design of the trial, SS was the project coordinator and involved in studies design, she was the Phase I clinical project manager and wrote the article. All authors read and approved the final manuscript.

## Supplementary Material

Additional file 1: Table S1 Main baseline demographic data of healthy male and female subjects (N = 72). This table shows the demographic data (median, min and max values) of the subjects included in the phase I trial by dose and flow rate. Click here for file
